# Chemical Diversity of Essential Oil of *Valeriana jatamansi* from Different Altitudes of Himalaya and Distillation Methods

**DOI:** 10.3390/molecules27082387

**Published:** 2022-04-07

**Authors:** Babit Kumar Thakur, Shivani, Mitali Mahajan, Probir Kumar Pal

**Affiliations:** 1 Division of Agrotechnology, Council of Scientific and Industrial Research-Institute of Himalayan Bioresource Technology, Post Box No. 6, Palampur 176061, India; babit.thakur.77@gmail.com (B.K.T.); shivanimanhas413@gmail.com (S.); mitalimahajan89@gmail.com (M.M.); 2 Academy of Scientific and Innovative Research (AcSIR), Ghaziabad 201002, India

**Keywords:** crop-ecology, essential oil, patchouli alcohol, seychellene, α-santalene, β-elemene

## Abstract

*Valeriana jatamansi* is an important temperate herb that is used in the pharmaceutical and essential oil industries. In India, this species is now on the verge of extinction due to the over-exploitation of its rhizomes from its natural habitat. It is hypothesized that the variations in bioactive compounds in its essential oil are very high among the wild populations as well as cultivated sources. Thus, this study was conducted to evaluate the chemical profiling of essential oil of four wild populations (Rupena, Kugti, Garola, and Khani) and two cultivated sources (CSIR-IHBT, Salooni), which were distilled at three consecutive days. The variation in oil concentration in roots/rhizomes was found significant (*p* ≤ 0.05), and the maximum value (0.35%) was registered with the population collected from Kugti and Khani. In essential oil, irrespective of population and distillation day, patchouli alcohol was the major compound, which ranged from 19 to 63.1%. The maximum value (63.1%) was recorded with the essential oil obtained from Garola’s population and distilled on the first day. The percentage of seychellene was abruptly increased with subsequent days of extraction in all the populations. The multivariate analysis revealed that the essential oil profiles of Rupena, Kugti, Garola, and CSIR-IHBT populations were found to be similar during the first day of distillation. However, during the second day, Rupena, Kugti, Khani, and CSIR-IHBT came under the same ellipse of 0.95% coefficient. The results suggest that the population of Kugti is superior in terms of oil concentration (0.35%), with a higher proportion of patchouli alcohol (63% on the first day). Thus, repeated distillation is recommended for higher recovery of essential oil. Moreover, repeated distillation can be used to attain *V. jatamansi* essential oil with differential and perhaps targeted definite chemical profile.

## 1. Introduction

*Valeriana jatamansi* Jones (Syn. *V. wallichii* DC.), commonly known as Indian valerian, belongs to the family of Valerianaceae; however, recently it has been categorized under Caprifoliaceae [1,2]. It is a small perennial medicinal herb that is naturally distributed in the temperate Himalaya between an altitude of 1000 and 3300 m. from masl [3,4]. In India and Nepal, *V. jatamansi* is widely used as a substitute for *V. officinalis*, which is mostly used in Europe [5]. *Valeriana jatamansi* has been widely used in the Indian Ayurvedic and Unani systems of medicine since ancient times [6]. Now, it has become the most acceptable sedative in modern medicine [6,7,8]. *Valeriana jatamansi* possesses a sedative property due to the presence of bioactive compounds such as ‘valepotriates’ in roots/rhizomes [9,10], which are non-glycosidic iridoid esters [11]. It is also used in Chinese traditional systems of medicine for its tranquilizing, hypnotic, and antiviral activities [12].

Importantly, the essential oil of *V. jatamansi* is now widely used in the flavor and fragrance industries, and about 30 pharmaceutical and essential oil-based products are commercially available [13]. Moreover, *V. jatamansi* has also been reported as a psychopharmacological agent [10,14,15] and is a highly effective against leprosy [16], epilepsy and hysteria [17]), nerve diseases [18], scorpion bite [19], cholera [20], anxiolytic properties [21], and curing lewy body dementia [22]. However, the present study has been emphasized only on essential oil content and the composition of different populations of *V. jatamansi* from the western Himalayan region in India.

In Himachal Pradesh, a Himalayan state of India, this plant is naturally grown on a moist slope of hills in the district of Chamba. However, a wide diversity of *V. jatamansi* exists in nature in this region [23]. Now in India, this species is at the edge of extinction due to the over-exploitation of its roots/rhizomes from its natural habitat to meet the burgeoning industrial demand. The industrial demand for this plant is increasing day by day for phytopharmaceutical as well as essential oil-based products. Since the major portion of the *V. jatamansi* is contributed through wild collections, there are major concerns about a consistent supply of quality raw material. Moreover, the collectors do not follow the standard protocol for harvesting to maintain the quality of essential oil. Essential oil of *V. jatamansi* is a complex mixture of volatile molecules, including monoterpenes and sesquiterpenes. More than 290 compounds have been identified in the essential oil of *V. jatamansi* so far [8]. The major compounds of *V. jatamansi* oil are patchouli alcohol, β-patchoulene, α-patchoulene, seychellene, α-selinene, and δ-guaiene. The relative proportion of major compounds determines the quality of the essential oil. The content of essential oil in roots/rhizomes varies largely with a range from 0.05–2.00% [24,25,26,27,28,29]). 

The variations in oil concentrations in roots/rhizome, as well as the percentage share of different compounds, are largely influenced by seasonal variations [30], populations [28], plant parts [4,27], geographical regions [31,32], type of materials [29], and genetic makeup [4,32,33]. The variations in secondary metabolites of *V. jatamansi* due to habited and altitude differences have also been reported [34]. The differences between wild and cultivated source of *V. jatamansi* in terms of essential oil content and antioxidant activities have also been noticed by Bhatt et al. [35]. The variations in chemical compositions of essential oil of *Rosa damascena* have been noticed due changes of ambient temperature and humidity during the time of flowering [36]. Since *V. jatamansi* is widely found in nature in the Chamba region, there is a possibility of large variations in oil content and compositions among its populations. However, the information about the variety, chemotype, or population that occurs in this region is limited. Farmers collect/harvest roots/rhizomes from the wild without having the information about chemical profiles. Farmers also use wild populations as planting material for cultivation in under-utilized land, or in orchards as an intercrop.

Another important issue with *V. jatamansi* is distillation methods, which largely influence the oil recovery and quality of essential oil. Generally, farmers distil their harvested roots/rhizomes in a commercial hydro-distillation unit after partial drying. Both dry and fresh roots/rhizomes are used for oil extraction, and the variations in oil concentration between fresh and dry materials have been reported [29,35,37]. Among the distillation conditions, time/duration is a key factor for the composition of essential oilssuch as pine (*Pinus ponderosa* Dougl. ex Laws) and Sweet Sagewort (*Artemisia annua* L.) [38,39], Japanese cornmint (*Mentha canadensis* L.) [40], lemongrass (*Cymbopogon flexuosus* Steud.) and palmarosa (*Cymbopogon martini* Roxb.) [41]. Moreover, the effect of distillation time on recovery of essential oil of *V. jatamansi* has been reported [29,42,43]. However, information regarding the effects of repetitive distillation on oil recovery and the quality of the oil is missing. It has been reported that essential oil fractions with specific chemical profiles can be managed through altering the time of hydro-distillation [44]. In case of rose essential oil, higher methyl eugenol concentration is noticed with long-term fermented flowers [45].

We hypothesized that repetitive distillation would be effective to increase the oil recovery and modification of the composition of the oil. It is a fact that the pH level of water influences the hydrolytic reactions during hydro-distillation. Seeing the above issues, there is a pressing need to understand the best population and effective distillation method for higher oil yield and desire quality. This experiment, therefore, is conducted to (i) investigate the profile of essential oil of different populations of *V. jatamansi* and (ii) to understand the effects of repetitive distillation on recovery and quality of essential oil.

## 2. Results

### 2.1. Essential Oil Content

**Figure 1:**
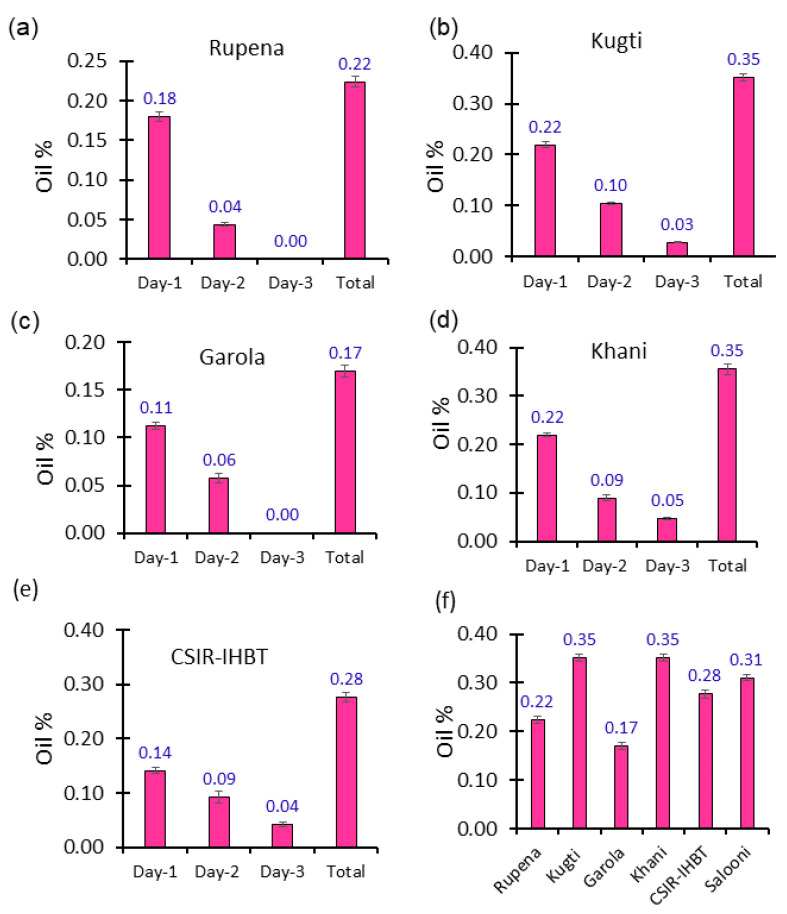
Concentration of essential oil (%) in the different populations of *V. jatamansi* collected from Rupena (**a**), Kugti (**b**), Garola (**c**), Khani (**d**), and CSIR-IHBT (**e**) during different distillation cycles, and total recovery of essential oil (%) of four wild populations (Rupena, Kugti, Garola, and Khani) and two cultivated sources of CSIR-IHBT and Salooni (**f**) under repetitive distillation method.

Analyzed data revealed that essential oil of *V. jatamansi* was obtained up to the third day of distillation; however, in the case of Rupena and Garola, oil was not obtained (Figure 1a–e) on the third day. Irrespective of sources of roots/rhizomes, the recovery rates (%) were higher on the first day of distillation, and thereafter the trends were declined. The total recovery (addition of 2/3 days’ samples) of essential oil (%) found significant (*p* ≤ 0.05) variations among the natural populations and cultivated source (Figure 1f). The populations collected from Kugti and Khani registered significantly (*p* ≤ 0.05) higher oil content (0.35%) than with other populations, a cultivated source at CSIR-IHBT, and oil obtained from the traditional extraction method. The lowest oil content (0.17%) was recorded with the population of Garola.

### 2.2. Composition of Essential Oil

**Figure 2:**
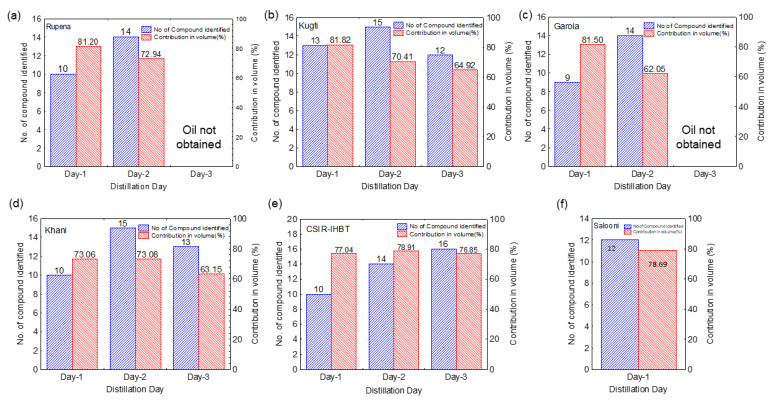
Number of volatile compounds identified and their contribution in volume (%) in the essential oil of *V. jatamansi* collected from Rupena (**a**), Kugti (**b**), Garola (**c**), Khani (**d**), CSIR-IHBT (**e**), and Salooni (**f**) under repetitive distillation method.

GC and GC–MS analysis data revealed that a maximum of 18 volatile compounds was identified from the essential oil obtained from hydro-distillation of *V. jatamansi* roots/rhizomes of different populations and cultivated sources (Figure 2). These compounds contributed up to 81.8% of the total volume (Figure 2) in this study. The highest number (16) of volatile compounds was identified with the cultivated source from CSIR-IHBT during third-day distillation (Figure 2e), whereas the lowest number (9) of compounds was identified with the population collected from Garola on day-one distillation (Figure 2c). However, the maximum (81.8%) and minimum (62.1%) sharing by the identified compounds in a total volume of essential oil was observed with the day-one distillation of the Kugti population and day-two distillation of Garola population, respectively (Figure 2b,c). In all populations, the numbers of identified compounds were higher in the essential oil extracted on the second day than on the first day, even these values were higher than the Salooni sample (traditional practice).

**Figure 3:**
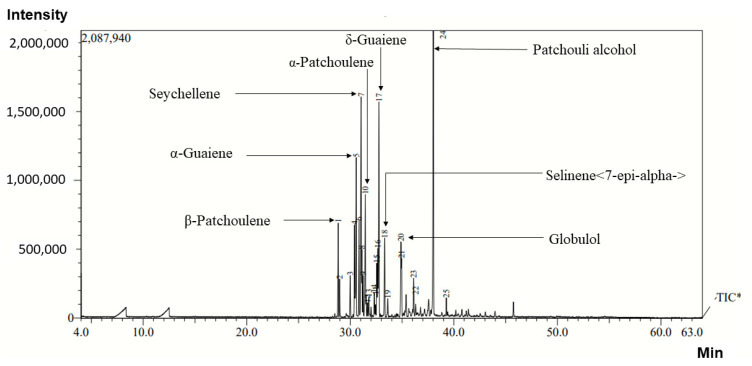
Representative GC–MS chromatograms of *V. jatamansi* oil sample.

The chemical profiles of the volatile oils from different natural and cultivated sources of *V. jatamansi* have been presented in Table 1. The data on compositions of essential oils revealed great variation among the different sources of material and distillation process. Irrespective of the source of materials and distillation day, the major compounds detected in the essential oil were patchouli alcohol, δ-guaiene, selinene<7-epi-alpha->, α-selinene, α-patchoulene, seychellene, and β-patchoulene, which have been illustrated in the representative chromatograms of essential oil in Figure 3. Patchouli alcohol was found to be the most abundant compound in all the essential oil obtained from different populations and distilled on different days (Table 1). Patchouli alcohol ranged from 19% to 63.1%. The maximum value (63.1%) was recorded with the essential oil obtained from Garola’s population and distilled on the first day, whereas the lowest value (19%) was recorded with the population collected from Khani and distilled on third day (Table 1). The essential oil of *V. jatamansi,* extracted through the traditional method (Salooni sample), registered maximum percentage of β-patchoulene (10.2%), α-guaiene (5.5%), α-humulene (5.0%), and δ-guaiene (7.3%) compared with the rest of the samples in this experiment. Humulene epoxide II was found only in three populations (Rupena, Garola, and Khani) during second-day distillation, and the maximum value (2.5%) was recorded with Garola’s population. Seychellene concentration was found to be at a maximum (13.3%) during the third day of distillation of the population collected from Kugti. Similarly, β–caryophyllene concentration was found to be at a maximum (3.8%) with Khani and CSIR-IHBT populations during the third day of distillation (Table 1).

**Figure 4:**
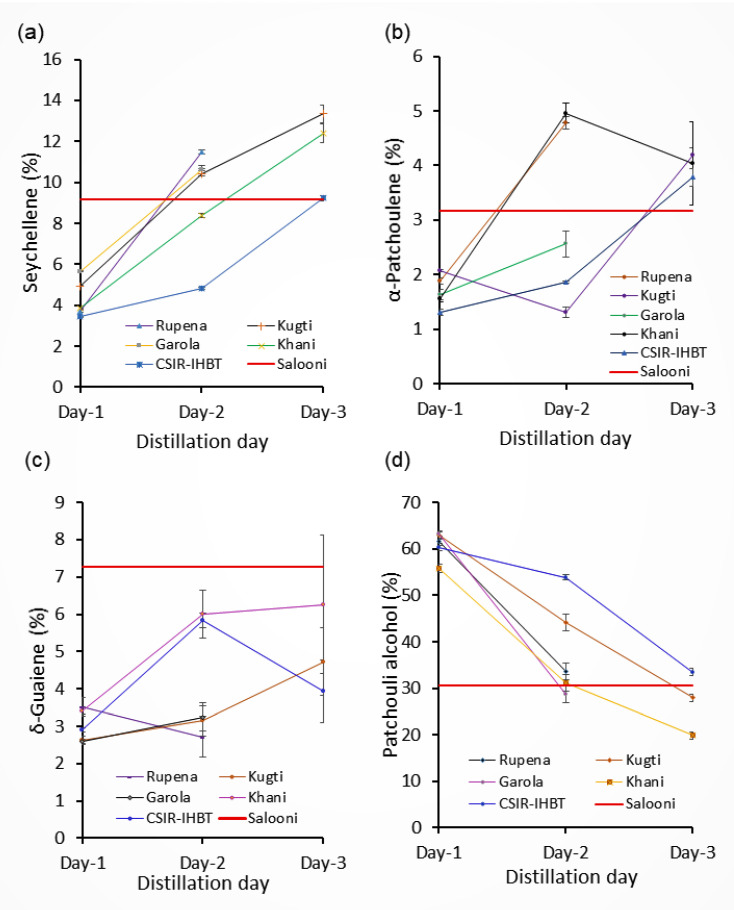
The dynamic of patchouli alcohol (**a**), seychellene (**b**), δ-guaiene (**c**), and α-patchoulene (**d**), under repetitive distillation method for the different populations.

The dynamic of important compounds in the essential oil of *V. jatamansi* populations has been influenced by the distillation days (Figure 4). The results indicated that maximum percentages of patchouli alcohol, irrespective of the source of materials, were registered with the oils obtained during the first day of distillation (Figure 4a). The patchouli alcohol concentrations in the oils obtained during the first day of distillation, irrespective of populations, were found to be substantially higher than the oil obtained from the traditional distillation method (Salooni). However, the concentrations of patchouli alcohol sharply declined during subsequent days of extraction (Figure 4a). In contrast, the percentage of seychellene was abruptly increased with subsequent days of extraction in all the populations (Figure 4b). The concentration of δ-guaiene in the essential oil of *V. jatamansi* was increased with second and third-day samples of Kugti, Garola, Khani, and CSIR-IHBT populations (Figure 4c). However, the trend of α-patchoulene concentration was not found consistently across the populations (Figure 4d). 

### 2.3. Principal Component Analysis (PCA)

**Figure 5:**
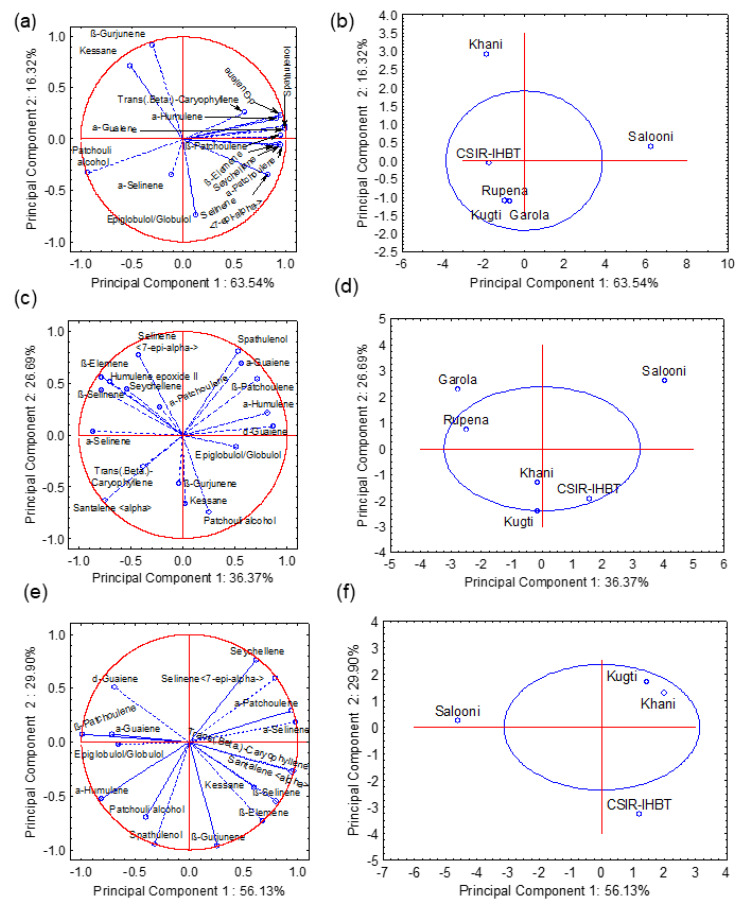
Principal component analysis of chemical compositions of essential oil in the different populations of *V. jatamansi* during first day (**a**,**b**), second day (**c**,**d**), and third day (**e**,**f**) of distillation.

The multivariate analysis in terms of PCA was performed to explore the relationship among the volatile compounds in the essential oil of *V. jatamansi* populations and to understand how these populations were different from each other. The compounds of the essential oil were treated as dependent variables. The separate PCAs that were constructed for all three days of distillation revealed that the first two components, PC1, and PC2, jointly explained 79.86, 63.06, and 86.03% of the total variations for first, second, and third-day distilled samples, respectively (Figure 5a–f). The eigenvalues of these two PCs are 9.53 and 2.45, respectively. The relationships among the compounds of essential oil and the magnitude of their contributions in the space of PC1 and PC2 have been presented in Figure 5a, c, and e for the first, second, and third day, respectively. For first day sample, PC1 has positive coefficients with β-patchoulene, β-elemene, α-guaiene, seychellene, α-humulene, α-patchoulene, δ-guaiene, selinene<7-epi-alpha->, and spathulenol; the loading values of these compounds were quite high (>0.83). Hence, it is clear that these eight compounds are highly correlated with each other (Figure 5a). However, PC1 has a negative coefficient with patchouli alcohol, with a loading value of −0.94. The PCA bi-plot of the first-day sample indicated that there were three distinct clusterings among the populations and cultivated sources. The populations from Kugti, Garola, and Rupena cultivated source of CSIR-IHBT were placed in the negative coordinate of both PCs, and all four samples came under the same ellipse of 0.95% coefficient (Figure 5b). The population of Khani and Salooni created independent groups. 

During the second day of distillation, β-patchoulene, α-guaiene, α-humulene, and δ-guaiene were situated in the positive coordinate of PC1, with loading values of 0.81, 0.56, 0.81, and 0.87, respectively (Figure 5c). Compounds such as β-elemene, α-santalene, β-selinene, α-selinene, and humulene epoxide II were also separated by the PC1 and placed in the negative coordinate with loading values of 0.78, 0.75, 0.78, 0.87, and 0.71, respectively. On the third day of distillation, the compounds were separated by both PCs, but there was no distinct cluster among the compounds (Figure 5e). The PCA bi-plots of second and third days of distillation also indicated three distinct groups among the natural populations and cultivated sources (Figure 5d,f). For the second-day sample, Kugti, Khani, and CSIR-IHBT fall under the same group whereas CSIR-IHBT is separated from Kugti and Khani for the third-day sample. The oil from Salooni did not form any group with the rest of the populations in this study. 

### 2.4. Physico-Chemical Properties of Soil of Naturally Distributed Locations of Valeriana jatamansi

The physico-chemical properties of the soil of different sample collecting locations have been presented in Table 2. The soil texture of the different locations ranged from sandy loam to silty clay. Sandy loam soil was found in Rupena and Salooni, whereas loamy soil was observed at Khani and Kugti. The soil of Palampur was heavy (silty clay). The significant (*p* ≤ 0.05) differences in terms of chemical properties (pH, EC, and OC, and available N, P, and K) of the soil were found among the locations. The highest pH (8.85) of soil was registered at Kugti, which was significantly (*p* ≤ 0.05) different from the rest of the locations. The lowest pH value (5.59) was registered with the soil collected from Palampur. The EC values of soils from different locations were also found to be significantly different (*p* ≤ 0.05), and the highest and lowest values were recorded in Garola and Palampur, respectively. Soil OC ranged from 0.71% at Garola to 3.34% at Salooni (Table 2). The bulk densities of soil collected from Kugti, Khani, Garola, and Palampur were found to be almost similar value (~1.48 g cm^−3^), but this value was significantly (*p* ≤ 0.05) higher than the values recorded from Rupena (1.25 g cm^−3^) and Salooni (1.34 g cm^−3^). Significant (*p* ≤ 0.05) variations in available N, P, and K content in soil were found among the locations. Maximum N and K contents were registered in the soil of Rupena, whereas maximum P was recorded with soil from Salooni (Table 2). Thus, it is evidenced from the result that the *V. jatamansi* is grown in a wide range of soil types with different nutritional statuses. Analysis of variance outcomes for determining the effect of location on oil concentration in different populations of *V. jatamansi* and on soil properties has been presented in Table 3. The values of mean squares with corresponding level of significant are presented. 

## 3. Discussion

In this study, significant variations were found in the essential oil concentrations, the main attribute of *V. jatamansi*, among the naturally distributed populations in Chamba region and cultivated sources. The highest oil concentration (0.35%) was registered with the population collected from Kugti and Khani. These differences among the populations were probably due to variations in micro-climatic conditions, soil characteristics, and genetic make-up. The altitude of these two locations is higher. Although the physico-chemical properties of the soils were found to differ among the locations, no definite correlation was found between soil characteristics and essential oil concentration. Despite the higher altitude, the lowest concentration of oil (0.17%) was registered with the population from Garola. This location wass characterized by higher EC (1.07 dS m^−1^) and relatively low OC (0.71%) and available N (61.12 kg ha^−1^), which might be another cause for the low concentration of essential oil in roots/rhizomes. Crop-ecological factors such as light, ambient temperature, and soil nutrient availability control the biosynthesis of essential oil [46]. In the mountainous region, variation in micro-climate is common, and this variation influences the qualitative and quantitative parameters of medicinal and aromatic plants [47,48,49]. The variations in essential oil concentrations in the wild populations and cultivated populations of *V. jatamansi* have also been reported earlier [27,35,43]. Despite proper agronomic practices, the oil concentration in the cultivated population (CSIR-IHBT) was lower than the populations from Kugti and Khani. This result was probably due to altitudinal variations and chemotype differences. The populations difference in terms of oil content has been reported from Himachal Pradesh, and the major chemical constituents and essential oil content are negatively correlated with altitude [32]. The low percentage of patchouli alcohol with the population from Khani during first cycle of distillation was noticed, probably due to the edaphic factor, particularly soil-available K content. Patchouli alcohol is synthesized in various stages through cis- farsenyl pyrophosphate [50]. The variations in essential oil composition of *V. jatamansi* due to growing conditions and location have also been reported by Rawat et al. [43] and Das et al. [51]. Thus, the population having higher amount of patchouli alcohol could be used for mass multiplication for commercial cultivation. This also suggested that variation in oil quality can be reduced by collecting the raw material from the cultivated sources.

Despite 6 h of distillation on the first day, some quantities of essential oil of *V. jatamansi* were obtained up to the third day of distillation from the populations collected from Kugti and Khani and from cultivated sources (CSIR-IHBT). The populations of Rupena and Garola also produced oil on the second day. These results were due to the fact thatsome compounds of essential oil of *V. jatamansi* such as β-elemene, α-santalene, and seychellene were not removed from the hypodermal layer in the root cortex region. The outcome is noteworthy, and the result can be used to maximize the recovery of essential oil of *V. jatamansi*. The effects of duration of distillation time on recovery of essential oil have been reported in many aromatic plants [38,39,41,44,52,53,54,55,56].

In this study, maximum of 18 volatile compounds were identified, which contributed up to 81.8% of the total volume (Figure 2), and the maximum number (16) of volatile compounds were identified with the cultivated source from CSIR-IHBT during third-day distillation (Figure 2e). This result could be due to the fact that the plants at CSIR-IHBT were grown with proper agronomic practices, which ultimately facilitated better conditions for the synthesis of a large number of compounds compared with other naturally distributed populations. The variations in genetic makeup among the populations may be another reason for changing the chemical profile of essential oil. On the other hand, the minimum number of volatile compounds were identified in the oil obtained at first-day distillation from the Garola population (Figure 2c). The variations in the number of compounds in the essential oil of *V. jatamansi* have been reported in the literature [4,25,30,57]. Thus, it is established from the present study that, irrespective of populations, the number of compounds has been increased either on the first day or on the second day of distillation (Figure 2a–f).

The variations in compositions of essential oils were found to be noticeable due to populations and distillation methods (Table 1). The differences in crop ecology may be another cause of variations in compositions of essential oil among the populations. In this study, variations in oil characteristics and altitudinal were observed (Table 1 and Table 4). The variations in essential oil composition from the different parts of India have been reported in *V. jatamansi* [27,35,42,51]. Patchouli alcohol, the most abundant compound in all the populations, was found to have noticeable differences, and the maximum concentrations were recorded on the first day of distillation, irrespective of populations (Table 1). The essential oil with different in fractions was probably due to boiling point, degree of solubility, molecular weight, and polarity of the compounds. The extraction of polar compounds during hydro-distillation is easier than that of terpene hydrocarbons [58].

The concentration of patchouli alcohol in Salooni (distilled in traditional method) was substantially lower than the rest of the populations distilled on the first day. This could be due to the fact that patchouli alcohol was lost during the drying and storage or post-harvest practices. Low concentrations of patchouli alcohol were also found in the second- and third-days’ oil for all populations. This result could be due to the fact that hydrolytic reactions occurred in the remaining hydrolat, leading to the breakage of the functional group in patchouli alcohol.

However, the higher concentrations of β-patchoulene, α-guaiene, α-humulene, and δ-guaiene were found in the Salooni sample probable due to the generation of more heat during the traditional distillation method. Disproportionation, as well as the cyclization process, occurs in monoterpenes at elevated temperatures [59]. Moreover, at high temperatures, compounds are formed through termination reactions [60]. Moreover, the higher concentration of humulene epoxide II (2.5% on the second day) makes difference to Garola from other populations.

Interestingly, the concentrations of seychellene were abruptly increased with subsequent days of extraction for all the populations. The use of seychellene compound as a non-selective candidate for inhibitor cyclooxygenase on pre-osteoblast cells has been reported [61]. The α-santalene, generally used in cosmetic, perfumery, and aromatherapy industries [62], was found only in second and third days. The α-santalene is the precursor of α-santalol, which is the main component of sandalwood oil from eastern India [63]. Similarly, β-elemene was found in the second- and third-days’ oil. β-elemene is a novel anticancer agent that is also reported for antitumor activity [64]. The improvement and/or generation of the new compound during the second and third days was probably due to oxidation, thermal degradation, chemical degradation, and (eventually) chemical conversion. This finding confirmed that the distillation method can be used to obtain the higher quantity of essential oil of *V. jatamansi* with variance chemical profiles. For example, if a high-patchouli alcohol oil is desirable, 6 h distillation is required for a single day. Similarly, if high concentrations of seychellene, α-santalene, or β-elemene are desirable, roots/rhizomes of *V. jatamansi* need to be distilled for second and/or third times. 

The results of this study prove the hypothesis that crop-ecology, source of materials, and distillation method determine the yield and composition of the essential oil of *V. jatamansi*.

## 4. Materials and Methods

### 4.1. Study Material

The fresh rhizomes with fibrous roots samples of four populations of *V. jatamansi* were collected from four different locations of Chamba district of Himachal Pradesh, India, which fall under the western Himalayan region. One sample was collected from a cultivated source from an Institutional experimental farm (Council of Scientific and Industrial Research-Institute of Himalayan Bioresource Technology, Palampur, India). The cultivated plant material was harvested at the age of two years. However, all the samples were collected during September. Since the wild populations were not distributed evenly, the samples were collected from about 20–30 m^2^ area in each location in three replicates. The population density was not uniform for all the locations. Harvested rhizomes and fibrous roots were washed with running tap water, and excess water was removed with the help of blotting paper. After recording the fresh weight of roots/rhizomes, samples were placed in air-tight polyethylene bags and brought to the laboratory for extraction of essential oil. The same procedures were followed for the cultivated sample from the Institutional farm. Besides, one oil sample was collected in three replicates from the farmer, which was extracted through a commercial hydro-distillation unit after partial drying of the roots/rhizomes in the Salooni region (Chamba). This is the common practice followed by the growers. This sample is also considered as a cultivated source. The collected oil sample was placed in an ice bag for further analysis. Geophysical positioning of the sampling locations was recorded with the help of the Garmin-eTrex 30x GPS, and the details are presented in Table 4.

### 4.2. Soil Collection and Analysis

The soil samples were collected from all locations, where fresh roots/rhizomes were collected. The soil samples were collected up to a depth of 15 cm after removing the litter from the surface. The soil samples were also collected in three replicates from the plant sampling area. Then, these soil samples were brought to the lab for further analysis. The collected soil samples were dried under shade and then ground with pestle and mortar, passing through the sieve of spacing 2 mm. Prepared samples were used for the analysis of pH, electrical conductivity (EC), soil organic carbon (SOC), available nitrogen (N), available phosphorus (P), and available potassium (K). For determination of pH and EC value of the collected sample, the soil water suspension in the ratio of 1:2 was prepared as per standard method described by Jackson [65]. Then, the pH and EC of soil water suspension (1:2) were determined with the help of pH meter (model Eutech Instruments pH 510) and EC meter (model Century), respectively. The soil OC was analyzed by the wet oxidation method [66]. Available N, P, and K were estimated as per standard methods reported in the literature [67,68].

### 4.3. Essential Oil Extraction

The fresh samples were chopped into small pieces and placed in 5L flasks. The sample size for all populations was 400 g, and the samples included only roots and rhizomes. The size of the rhizome was upto 15 cm. The ratio between water and fresh roots/rhizomes was 2:1 (*v/w*) during distillation in laboratory conditions. The oil was extracted through the hydro-distillation process in a Clevenger-type apparatus for 6 h. After the collection of oil, the Clevenger apparatus was left as it was without removing the water and roots/rhizomes for re-distillation. The next day, the respective Clevenger apparatuses were run again without changing the water for 6 h, and the oil recovery was recorded and collected separately. The same procedure was repeated on the 4th day; however, oil was received up to 3rd day. Thus, 4th day was excluded from the experiment. The amount of oil extracted was recorded daily, and the percentages of oil recovery (*v/w*) for each cycle were calculated based on the plant material used at first day. The total oil yield and oil percentage were calculated by adding the entire three days’ recovery. Then, the extracted oil was dehydrated over anhydrous sodium sulfate (Merck) and stored in sealed glass vials at 4 °C for further studies.

### 4.4. GC–MS Analysis 

The GC–MS analysis of essential oil of *V. jatamansi* was done by a QP2010 (Shimadzu Corp., Tokyo, Japan) GC–MS system, which was fitted out with an AOC 5000 Auto injector and a ZB-5 (SGE International, Ringwood, VIC, Australia) silica capillary column with a dimension of 30 m × 0.25 mm i.d., and 0.25 µm film thickness. The temperature of the oven was programmed in such a way that started from 70 °C (for 4 min) to 220 °C with a slope of 4 °C min^−1^ and detention time of 5 min. The fixed temperatures for injector and interface were 240 °C and 250 °C, respectively. The flow rate of helium, a carrier gas, was 1.05 mL min^−1^, and the voltage for ionization was 70 eV.

### 4.5. GC Analysis

GC analyses of essential oil of *V. jatamansi* were carried out by a Shimadzu GC-2010 gas chromatograph (Shimadzu, Tokyo, Japan) that was attached with a flame ionization detector (FID) and a ZB-5 MS capillary column (30 m × 0.25 mm, fused silica, and film thickness 0.25 µm). The temperature of the oven was programmed at 70 °C for 3 min and gradually increased up to 220 °C with a slope of 4 °C min^−1^ and detention time of 5 min. The temperatures for injector and interface were 220 °C and 250 °C, respectively. Then, the individual compounds were quantified based on the peak-area percentage of the chromatogram.

### 4.6. Identification of Components

The different compounds of essential oil of *V. jatamansi* were identified through using retention indices (RI) that were computed with reference to homologous series of n-alkanes (C8–C24). The compounds were confirmed through a comparison between RI and mass spectra with the National Institute of Standards and Technology mass spectral (NIST-MS) database [69]. The GC and GC–MS analyses were conducted for all three replications and oil obtained at all rounds of extraction. The total percentage shares of compounds were computed by addition of the individual percentage of all identified compounds.

### 4.7. Experimental Design and Statistical Analysis

The data obtained from the four populations from wild and one cultivated samples were subjected to analysis of variance (ANOVA). Differences among the populations in terms of total oil concentration were assessed with the Fishers least significant differences (LSD) test values only when the *F-test* in ANOVA was found to be significant (*p* = 0.05). In case of soil parameters, Fishers LSD *post-hoc test* was applied to compare the means. Principal component analysis (PCA) was performed to categorize the populations based on chemical compositions of essential oil. The PCA was conducted separately for all three types of oil samples, which were obtained from three different distillation days. However, a sample received from traditional practice was included in all the PCA for comparative studies. All the ANOVA and PCA were performed with the help of statistical software (Statistica 7 software; Stat. Soft Inc., Tulsa, OK, USA). The data on compounds of essential oil of this study were presented as mean ± standard error (SE) since many compounds were not detected during different distillation cycles. 

## 5. Conclusions

The results of the present study elucidate that yield and composition of essential oil of *V. jatamansi* is largely governed by the crop-ecology and distillation methods. Our results also confirm that different chemo-types exist in natural populations in Chamba (H.P., India). The total recovery of essential oil (%) was found to have significant variations (*p* ≤ 0.05) among the source of materials, and the maximum value (0.35%) was recorded with the populations from Kugti and Khani. All the populations are characterized by being rich in patchouli alcohol. This study also confirmed that the repetitive distillation method can be used to obtain a higher quantity of essential oil of *V. jatamansi* with variance chemical profiles. The concentrations of some compounds such as seychellene, α-santalene, or β-elemene can be increased through repetitive distillation. It can also be concluded that the species *V. jatamansi* has wide adaptability, and it can be commercially cultivated with an altitudinal range of 1354–2140 m. It can also be grown in sandy loam to silty clay soil, with a wide pH range of 5.59–8.85 and different nutritional levels. However, to discriminate between the effects of population and crop-ecology and to identify the elite material, all the collected and/or characterized populations should be evaluated under the same conditions.

## Figures and Tables

**Table 1:** The dynamic of composition of essential oil (%) in different populations of *V. jatamansi,* under repetitive distillation method.

Compound	Rupena		Kugti			Garola		Khani			CSIR-IHBT			Salooni
	Day-1	Day-2	Day-1	Day-2	Day-3	Day-1	Day-2	Day-1	Day-2	Day-3	Day-1	Day-2	Day-3	Day-1
β-Patchoulene	4.5 ± 0.33	3.8 ± 0.27	1.9 ± 0.09	-	-	1.9 ± 0.11	-	2.0 ± 0.07	2.5 ± 0.12	-	1.7 ± 0.01	2.0 ± 0.08	-	10.2 ± 0.05
β-Elemene	-	1.2 ± 0.00	0.4 ± 0.01	0.7 ± 0.03	1.5 ± 0.03	-	1.3 ± 0.16	-	0.9 ± 0.02	1.7 ± 0.06	-	0.7 ± 0.02	2.9 ± 0.14	0.8 ± 0.03
Santalene <alpha>	-	1.1 ± 0.04	-	1.2 ± 0.01	1.2 ± 0.02	-	0.8 ± 0.20	-	1.1 ± 0.02	1.5 ± 0.05	-	0.7 ± 0.02	1.7 ± 0.04	-
β-Gurjunene	-	-	-	0.9 ± 0.03	-	-	-	0.9 ± 0.02	2.8 ± 0.05	-	-	-	1.5 ± 0.03	-
α-Guaiene	1.7 ± 0.02	2.2 ± 0.14	1.8 ± 0.08	0.7 ± 0.02	1.1 ± 0.03	1.9 ± 0.05	1.2 ± 0.16	1.7 ± 0.02	2.9 ± 0.15	4.1 ± 0.24	1.9 ± 0.18	1.1 ± 0.03	2.3 ± 0.13	5.5 ± 0.29
Trans(.Beta.)-Caryophyllene	0.9 ± 0.01	3.0 ± 0.01	1.1 ± 0.01	2.3 ± 0.04	3.1 ± 0.08	-	1.6 ± 0.02	0.9 ± 0.02	2.5 ± 0.03	3.8 ± 0.22	1.3 ± 0.02	1.8 ± 0.04	3.8 ± 0.06	1.8 ± 0.03
Seychellene	3.7 ± 0.05	11.5 ± 0.10	4.9 ± 0.04	10.4 ± 0.39	13.3 ± 0.40	5.7 ± 0.01	10.6 ± 0.04	3.8 ± 0.03	8.4 ± 0.10	12.4 ± 0.47	3.4 ± 0.04	4.8 ± 0.03	9.2 ± 0.14	9.2 ± 0.11
α-Humulene	1.0 ± 0.02	-	0.8 ± 0.01	1.5 ± 0.15	-	2.3 ± 0.03	0.8 ± 0.05	1.7 ± 0.03	3.4 ± 0.34	1.2 ± 0.04	1.5 ± 0.02	1.3 ± 0.04	3.3 ± 0.46	5.0 ± 0.06
α-Patchoulene	1.9 ± 0.06	4.8 ± 0.12	2.1 ± 0.02	1.3 ± 0.09	4.2 ± 0.14	1.6 ± 0.09	2.6 ± 0.24	1.6 ± 0.06	4.9 ± 0.18	4.0 ± 0.76	1.3 ± 0.05	1.8 ± 0.02	3.8 ± 0.16	3.2 ± 0.00
β-Selinene	-	0.9 ± 0.03	-	0.6 ± 0.13	0.8 ± 0.04	-	1.9 ± 0.01	-	-	1.7 ± 0.11	-	-	2.2 ± 0.04	-
α-Selinene	-	3.2 ± 0.33	0.5 ± 0.09	0.8 ± 0.03	2.3 ± 0.20	-	2.3 ± 0.11	-	2.4 ± 0.04	2.3 ± 0.10	-	1.1 ± 0.00	1.7 ± 0.24	-
δ-Guaiene	3.5 ± 0.26	2.7 ± 0.55	2.6 ± 0.12	3.14 ± 0.41	4.7 ± 0.90	2.6 ± 0.06	3.2 ± 0.37	3.4 ± 0.09	6.0 ± 0.64	6.3 ± 1.86	2.9 ± 0.06	5.8 ± 0.20	3.9 ± 0.84	7.3 ± 0.23
Selinene<7-epi-alpha->	0.5 ± 0.10	3.1 ± 0.12	1.1 ± 0.02	1.0 ± 0.14	3.5 ± 0.06	1.4 ± 0.04	3.0 ± 0.01	-	1.5 ± 0.01	3.3 ± 0.10	-	1.9 ± 0.03	2.6 ± 0.07	2.2 ± 0.04
Kessane	-	-	0.4 ± 0.00	0.9 ± 0.06	-	-	-	1.1 ± 0.00	1.9 ± 0.07	1.2± 0.17	0.9 ± 0.02	0.4 ± 0.00	1.0 ± 0.03	-
Spathulenol	-	-	-	-	-	-	0.7 ± 0.02	-	-	-	-	-	2.8 ± 0.06	1.6 ± 0.08
Epiglobulol/ Globulol	1.8 ± 0.07	1.3 ± 0.03	1.2 ± 0.01	0.9 ± 0.06	1.1 ± 0.07	1.1 ± 0.09	-	-	-	-	1.9 ± 0.02	1.5 ± 0.04	0.7 ± 0.05	1.3 ± 0.01
Humulene epoxide II	-	0.7 ± 0.02	-	-	-	-	2.5 ± 0.04	-	0.6 ± 0.04	-	-	-	-	-
Patchouli alcohol	61.7 ± 0.49	33.6 ± 1.78	63.0 ± 0.91	44.1 ± 1.82	27.9 ± 0.77	63.1 ± 0.65	28.8 ± 1.85	55.8 ± 0.47	31.2 ± 1.05	19.83 ± 0.46	60.2 ± 0.63	53.8 ± 0.56	33.5 ± 0.68	30.7 ± 0.49

**Table 2:** Physico-chemical properties of soil of different locations of sample collection.

Location	Soil Texture	pH	EC _(25 °C)_ (dS m^−1^)	Organic Carbon (%)	Bulk Density (g cm^−3^)	Available N (kg ha^−1^)	Available P (kg ha^−1^)	Available K (kg ha^−1^)
Rupena (Chamba)	Sandy Loam	7.61	0.847	3.22	1.25	284.53	18.54	1231.83
Kugti (Chamba)	Loam	8.85	0.484	1.23	1.48	80.22	12.45	642.54
Garola (Chamba)	Silty Clay Loam	7.81	1.074	0.71	1.47	61.12	33.75	1044.37
Khani (Chamba)	Loam	6.67	0.411	2.05	1.48	139.29	14.76	185.38
CSIR-IHBT, Palampur	Silty Clay	5.59	0.057	1.35	1.48	77.47	13.90	295.98
Salooni (Chamba)	Sandy Loam	6.40	0.210	3.34	1.34	190.89	37.62	218.68
SEm (±)		0.12	0.00	0.04	0.03	7.25	1.45	19.35
CD (*p* = 0.05)		0.38	0.01	0.12	0.09	23.15	4.63	61.76

**Table 3:** Analysis of variance for determining the effect of location on oil concentration in different populations of *V. jatamansi* and on soil properties (values of mean squares are presented).

Source of Variation	df	Parameters Tested							
		Oil%	pH	EC _(25 °C)_ (dS m^−1^)	Organic Carbon (%)	Bulk Density (g cm^−3^)	Available N (kg ha^−1^)	Available P (kg ha^−1^)	Available K (kg ha^−1^)
Location	5	0.016 **	4.07 **	0.442 **	3.592 **	0.027 **	22,306.07 **	361.875 **	604,904.63 **
Error	12	0.00	0.042	0.00	0.003	0.002	157.491	6.529	960.476

df—degree of freedom; EC—Electrical conductivity of soil. ** *p* < 0.01.

**Table 4:** Geophysical positioning of different locations of sample collection.

Location	Altitude (m)	Latitude	Longitude
Rupena (Chamba)	1810	32°27′68″ N	76°04′54″ E
Kugti (Chamba)	2140	32°34′35″ N	72°27′82″ E
Garola (Chamba)	2003	32°25′11″ N	72°28′32″ E
Khani (Chamba)	2014	32°27′39″ N	76°29′1″ E
CSIR-IHBT, Palampur	1354	32°06′61″ N	76°33′77″ E
Salooni (Chamba)	2032	32°26′43″ N	76°32′10″ E

## Data Availability

Not applicable.

## Sample Availability

Samples of the essential oils are available from the authors.
